# An *in-situ* synthesis of Ag/AgCl/TiO_2_/hierarchical porous magnesian material and its photocatalytic performance

**DOI:** 10.1038/srep21617

**Published:** 2016-02-17

**Authors:** Lu Yang, Fazhou Wang, Chang Shu, Peng Liu, Wenqin Zhang, Shuguang Hu

**Affiliations:** 1 State Key Laboratory of Silicate Materials for Architectures, Wuhan University of Technology, Wuhan 430070, PR China; 2 School of Materials Science and Engineering, Wuhan University of Technology, Wuhan 430070, PR China; 3 School of Chemistry, Chemical Engineering and Life Science, Wuhan University of Technology, Wuhan 430070, PR China

## Abstract

The absorption ability and photocatalytic activity of photocatalytic materials play important roles in improving the pollutants removal effects. Herein, we reported a new kind of photocatalytic material, which was synthesized by simultaneously designing hierarchical porous magnesian (PM) substrate and TiO_2_ catalyst modification. Particularly, PM substrate could be facilely prepared by controlling its crystal phase (Phase 5, Mg_3_Cl(OH)_5_·4H_2_O), while Ag/AgCl particles modification of TiO_2_ could be achieved by *in situ* ion exchange between Ag^+^ and above crystal Phase. Physiochemical analysis shows that Ag/AgCl/TiO_2_/PM material has higher visible and ultraviolet light absorption response, and excellent gas absorption performance compared to other controls. These suggested that Ag/AgCl/TiO_2_/PM material could produce more efficient photocatalytic effects. Its photocatalytic reaction rate was 5.21 and 30.57 times higher than that of TiO_2_/PM and TiO_2_/imporous magnesian substrate, respectively. Thus, this material and its intergration synthesis method could provide a novel strategy for high-efficiency application and modification of TiO_2_ photocatalyst in engineering filed.

TiO_2_ photocatalyst has been extensively investigated and commercial application in several fields due to its high redox ability and stability under light illumination^1^,^2^,^3^,^4^. However, research on TiO_2_ photocatalysis suggested that its constructive and highly effective application remains a series of issues, of which the immobilized performance, visible light response and electron-hole recombination rate were considered to be its developing and application bottlenecks^5^,^6^. Recently, it was found that Ag/AgX (Br, Cl, I) coupling with TiO_2_ photocatalyst revealed a fascinating photocatalytic performance^7^,^8^,^9^,^10^,^11^,^12^,^13^. It has been proven that Ag nanoparticles (Ag NPs) can obtain a strong visible light absorption by its Surface Plasmon Resonance (SPR), while AgX/TiO_2_ structure is useful for photo-generated charges separation. Based on these principles, Lu *et al.*^14^ further enhanced the photocatalytic activity of Ag/AgCl/TiO_2_ photocatalyst by using transition metals as a co-catalyst. These reports demonstrated that various photocatalysts with high photocatalytic activity could be easily achieved by its surface modification and doping methods. Therefore, the main concern of these materials applied in practical engineering is seems to seek a reasonable immobilization substrate.

Some 3D interconnected hierarchical porous substrates including metal based, glass based, ceramics based, carbon based and cementitious materials, etc.^15^,^16^, could provide higher surface area and disperse ability for photocatalyst coating. Most importantly, these substrates may possess an excellent reagents diffusion ability to active sites located inside framework or inter-particulate, thus are beneficial for photocatalysis^17^. However, the production of these substrates usually needs to couple the specific templates or surfactants with high temperature treatment, which make them difficult to regulate. Moreover, the large scale and low cost synthesis methods of these substrates and their combination with the modified photocatalysts also require a further investigation. In that case, the integration design of photocatalysts modification and their coating substrates are very interesting and important for high efficiency photocatalysis and the actual engineering application.

Herein, we presented a new kind of Ag/AgCl/TiO_2_/hierarchical porous magnesian material, which can be conveniently synthesized by simultaneously designing the hierarchical porous magnesian (PM) substrate and Ag/AgCl modified TiO_2_ photocatalyst. Especially, owing to the physical structure characteristics of phase 5 (Mg_3_Cl(OH)_5_·4H_2_O) crystals, the prepared material offered a well gas diffusion and light transmittance channels for photocatalysis. Meanwhile, by using phase 5 coupling with Ag^+^ ion, the light absorption performance and charges transfer properties of prepared materials were further enhanced. Thus, this method and prepared material could provide a new strategy for high-efficiency application of TiO_2_ photocatalyst in actual engineering field.

## Results

**Figure 1:**
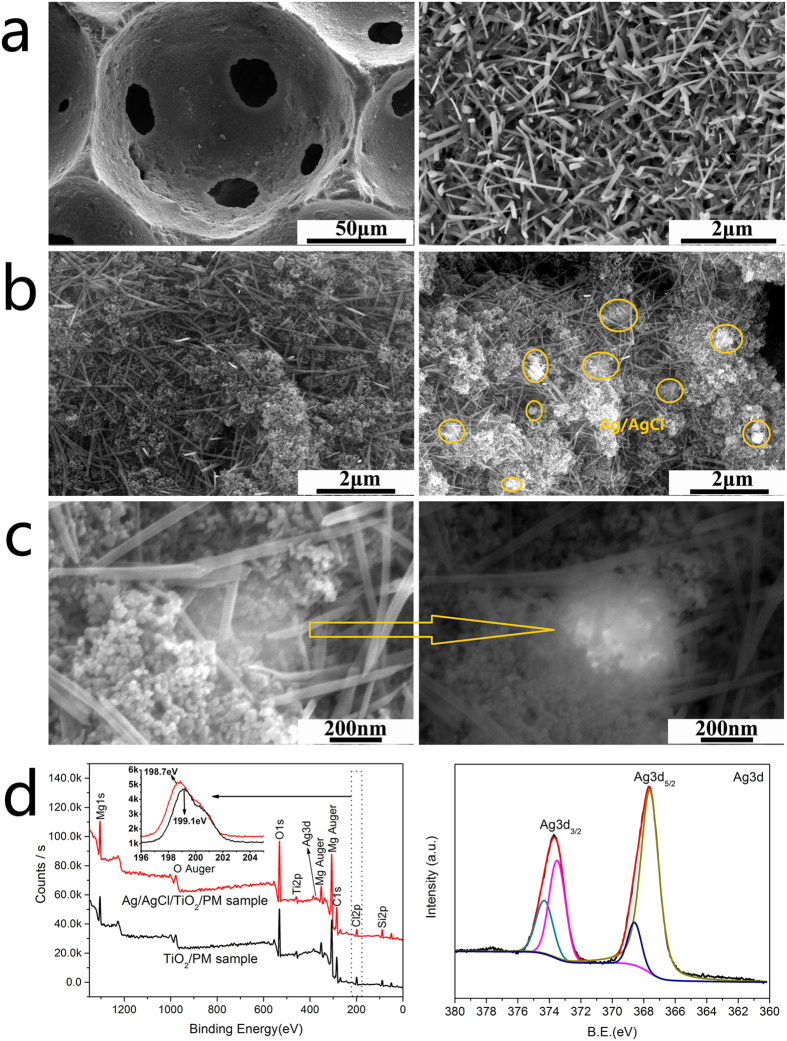
Characterization of porous magnesian (PM), TiO2/PM and Ag/AgCl/TiO2/PM samples. (**a**) Typical ESEM images of porous magnesian (PM) substrate. (**b**) Typical ESEM images of TiO_2_/PM and Ag/AgCl/TiO_2_/PM sample. (**c**) Typical high resolution ESEM and BSEM images of Ag/AgCl/TiO_2_/PM sample (2.34 × 10^−4 ^wt.% Ag content). (**d**) Typical XPS photographs of TiO_2_/PM and Ag/AgCl/TiO_2_/PM (2.34 × 10^−4 ^wt.% Ag content), the left image is survey pattern and Cl2p spectra (inset), the right image is high resolution XPS spectra image of Ag3d.

Owing to the air-solid interface reaction property of photocatalytic process, the substrate surface area, light transmission and gas absorption ability have significant influence on photocatalytic efficiency for supported photocatalytic materials^18^,^19^. Fig. 1a shows the ESEM images of porous magnesian (PM) substrate. It can be seen that PM substrate are composed of macro air pores, interconnection pores and interlaced network micro-pores, respectively. Interestingly, PM are almost composed by needle-like crystals (phase 5, Mg_3_Cl(OH)_5_·4H_2_O), and therefore forming the 3D network structure and interlaced network micro-pores^20^,^21^. After incorporating TiO_2_ particles, it can be clearly observed that the loose and interpenetration textures were formed between TiO_2_ particles and needle like phase 5 crystals (Fig. 1b), which are help for the absorption and photocatalysis of TiO_2_ particles. Meanwhile, the facile and low energy consumption synthesis route of PM substrate could be easily controlled in comparison to the high temperature preparation method of porous metal and ceramics. Based on the features of porous magnesia (PM) substrate component, Ag/AgCl semiconductor could be facilely formed by the ion exchange between AgNO_3_ and Phase 5 crystals (Mg_3_Cl(OH)_5_·4H_2_O). In Fig. 1b, it can be seen that Ag/AgCl particles were successfully formed. Furthermore, the high resolution ESEM and BSEM images of Ag/AgCl/TiO_2_/PM sample (Fig. 1c) indicated that TiO_2_ particles, Ag/AgCl particles and needle like phase 5 crystals have an excellent connectivity and combination structure.

XPS photographs were employed to further demonstrate the surface elemental compositions and chemical status of Ag/AgCl/TiO_2_/PM photocatalytic material, as shown in Fig. 1d. The typical XPS survey pattern of TiO_2_/PM mainly illustrated the XPS spectra of Mg, Cl, O, Ti and C elements, however, compared to TiO_2_/PM sample, an extra Ag element peak was observed in Ag/AgCl/TiO_2_/PM sample. Moreover, the high resolution XPS spectra of Cl2p (198.7 eV, inset in Fig. 1d) obviously shift for Ag/AgCl/TiO_2_/PM sample compared to TiO_2_/PM sample (Cl2p = 199.1 eV), indicating the bound Cl^−^ of phase 5 was partially deprived by Ag^+^ for forming the more stable AgCl precipitation. The Ag3d peak can be deconvoluted into two sets of double peaks, of which Ag3d_3/2_ peak (373 eV) was divided into two different peaks at 373.6 and 374.3 eV, whereas Ag 3d_5/2_ peak (367 eV) was divided into 368.6 and 367.5 eV. According to the reports of Zhang^22^ and Huang^23^, the peaks at 373.6 and 367.5 eV were attributed to metallic silver binding energy in Ag3d_3/2_ and Ag 3d_5/2_, and the peaks at 374.3 and 368.6 eV were ascribed to the Ag3d_3/2_ and Ag 3d_5/2_ of Ag^+^ in AgCl, indicating the Ag/AgCl had successfully been modifyed on TiO_2_/PM sample.

**Figure 2:**
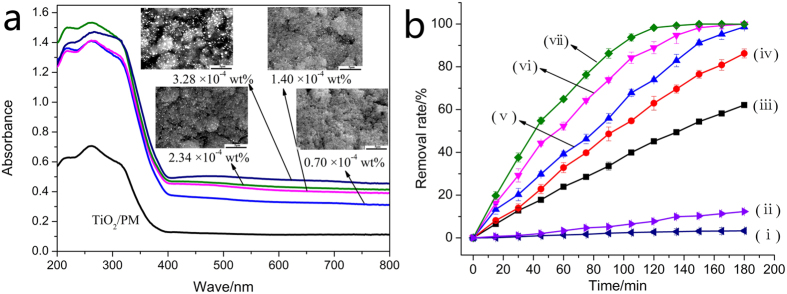
Comparative light absorption ability and photocatalytic performance of various samples. (**a**) UV–vis diffuse reflectance spectra of TiO_2_/PM and Ag/AgCl/TiO_2_/PM with various Ag modification contents ((0.7, 1.4, 2.34, 3.28) × 10^−4 ^wt.%), the inset images are corresponding BSEM images of Ag/AgCl/TiO_2_/PM samples. (**b**) Photocatalytic removal of benzene by various samples: (i) No catalytic; (ii) TiO_2_/imporous magnesian sample; (iii) TiO_2_/PM sample; (iv) ~ (v): Ag/AgCl/TiO_2_/PM sample with (0.7, 1.4, 2.34, 3.28) × 10^−4 ^wt.% Ag content.

The BSEM image could well and exactly reflect the distribution and sizes of Ag/AgCl particles due to the enormous atomic coefficient difference between Ti and Ag. As shown in the inset of Fig. 2a, it is clear revealed that the bright Ag/AgCl particles became more and more on TiO_2_/PM surface with increasing Ag modification contents from 0.70 × 10^−4 ^wt.% to 3.28 × 10^−4 ^wt.%. Meanwhile, the particle sizes also presented an irregular increase, when the Ag modification content achieved in 3.28 × 10^−4 ^wt.%, the biggest Ag/AgCl particle exceeded 500 nm, this may be due to the self-aggregation effect of metallic silver. Fig. 2a also shows the UV–vis diffuse reflectance spectra of TiO_2_/PM sample and various Ag/AgCl/TiO_2_/PM samples. It can be seen that TiO_2_/PM sample shows a strong absorption in UV light region. Furthermore, compared to the TiO_2_/PM sample, Ag/AgCl modified samples not only show stronger light absorption in the range of 200–400 nm, but also have substantial absorption in visible region (380 ~800 nm). These may be caused by the direct and indirect band gap of AgCl (3.25 eV), and the SPR of Ag nanoparticles^24^. In fact, the unique SPR peaks of Ag nanoparticles on AgCl surface are greatly influenced by their shapes and diameters, therefore various Ag nanoparticles lead to broad SPR peaks, which covered a wide visible light absorption range, and this is in good agreement with inset BSEM images.

The prepared samples are expected to have excellent photocatalytic activity for the degradation of organic contaminants due to their intense gas and light absorption performance. Fig. 2b shows the photocatalytic degradation of gaseous benzene using various samples. It can be observed that the photocatalytic performance of modified material increased gradually with increasing Ag modification contents. More specifically, Ag/AgCl/TiO_2_/PM with 3.28 × 10^−4 ^wt.% Ag content processed the highest photocatalytic activity for the decomposing of gaseous benzene, its reaction rate (*k *= 2.36 × 10^−2 ^min^−1^) was 5.21 times and 30.57 higher than that of TiO_2_/PM and TiO_2_/ imporous magnesian substrate sample (Table 1), indicating that the PM subatrate micro structure and catalyst modification play important roles in enhancing the photocatalytic activity of TiO_2_ particles. On one hand, the features of PM substrate like abundant pores, perforated cell walls and interlaced network structure were investigated to be useful for increasing TiO_2_ coating areas, gas diffusion and light transmittance channels, therefore providing more reaction sites between TiO_2_ particles and gaseous molecules, and ultimately enhancing photocatalytic efficiency^20^,^25^. On other hand, TiO_2_ photocatalyst can be excited the photogenerated electron–hole pairs (e_0_^−^-h^+^) u-nder UV light, Ag nanoparticles on the surface of TiO_2_ also could absorb the UV light and partial visible light for generating the (e_1_^−^-Ag^+^) pairs. In that case, although partial e_0_^-^ could react with Ag^+^ to form Ag, a majority of e_0_^−^ and e_1_^−^ would transfer to the surface of photocatalyst due to the enhanced local electric-field intensity by the Ag/AgCl^26^,^27^. Therefore, the more photogenerated electrons could facilitate the trapping of O_2_ molecules for forming more superoxide ions (•O^2−^) and other oxygen species, which contributes to the improvement of photocatalytic performance. Meanwhile, the photogenerated holes (h^+^) could react with AgCl and OH^−^ to form Ag^+^, Cl^0^ and •OH, respectively, in which Cl^0^ and •OH could effective degrade organic pollutants, thus the photocatalytic performance could be further enhanced.

## Discussion

**Figure 3:**
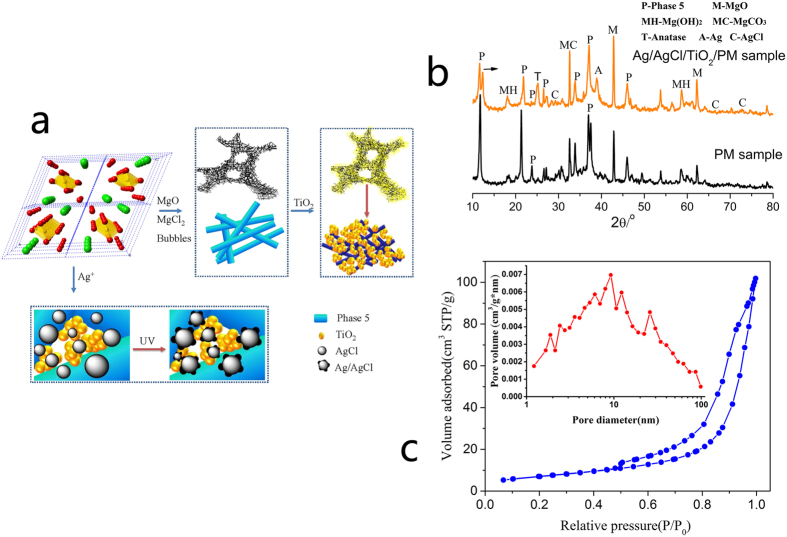
The *in situ* synthesis and photocatalytic performance mechanism analysis of Ag/AgCl/TiO2/PM sample. (**a**) The integration synthesis schematic of Ag/AgCl/TiO_2_/PM sample. (**b**) XRD patterns of PM sample and Ag/AgCl/TiO_2_/PM sample with 2.34 × 10^−4 ^wt.% Ag content. (**c**) Typical N_2_ adsorption–desorption isotherms and pore size distribution of TiO_2_/PM sample.

It is very interesting and meaningful to investigate the potential integration synthesis and catalytic mechanism of Ag/AgCl/TiO_2_/PM photocatalytic materials. It is well know that the main conditions and steps of photocatalytic reaction include: (1) interface contact and absorption between photocatalyst and pollutants; (2) appropriate light energy irradiating on photocatalyst surface; (3) pollutants be decomposed on photocatalyst surface; (4) desorption of decomposition products on photocatalyst surface. Among them, it is clear that the absorption ability and photocatalytic activity of photocatalytic materials play important roles in improving the removal effects of pollutants. In this study, the phase 5 as a main hydration product of PM substrate, is composed with triple chains octahedral (one Mg(OH)_6_ and two Mg(OH)_3_(OH_2_)_3_ or Mg(OH)_4_(OH_2_)_2_ octahedra along axis b infinite extension) and the chains of intercalating Cl atoms and water molecules (SOF = 0.500, Fig. 3a), which means that phase 5 structure could be controlled by Mg-O octahedral amounts^28^. Thus, as shown in Figs 1a and 3a,b, through introducing bubbles and controlling the mixture ratios of MgO and MgCl_2_ solution, the amounts of Phase 5 and its accumulation structure could be regulated easily. Furthermore, by using the negative pressure coating method, TiO_2_ particles could to be filled into phase 5 interlaced network pores (Figs 1b and 3b), which would obviously improve the contact areas between TiO_2_ particles with light energy and pollutants. As shown in Fig. 3c, the isotherm of TiO_2_/PM sample provided more specific results about those inference. It can be seen that the isotherm of TiO_2_/PM sample had one hysteresis loop at relative pressure between 0.43 and 1, demonstrated that it was mesoporous structures. Particularly, in 0.9~1.0 high pressure regions, the absorption volume rising faster indicated that the material contained macro pores. The inset pore size distribution curves also revealed that TiO_2_/PM had an obvious pore peaks in the range of 1~100 nm. Those could offer the gas diffusion and light transmittance channels from the particle surface to the interior, which are expected to be useful in the photocatalytic process.

More importantly, as shown in Fig. 3a,b, the Cl ion resource of PM substrate could simultaneously provide the effective and facile modification method for the formation of Ag/AgCl semiconductor, which was proved by the results of Fig. 1b–d, means the light absorption performance and charges transfer properties of TiO_2_ particles could be enhanced by Ag/AgCl/TiO_2_ composite structure. Furthermore, as shown in Fig. 2a, it also can be seen that the *UV-Vis* spectral data for the Ag/AgCl/TiO_2_/PM sample (3.28 × 10^−4 ^wt.% Ag content) shows higher absorption from *UV* to *Vis* light regions compared to other controls. Overall, based on PM substrate special physical structure coupling with its chemical *in situ* Ag/AgCl modification method to TiO_2_ particles, the high-activity Ag/AgCl/TiO_2_/PM photocatalytic composite material was explored, which shown the higher photocatalytic efficiency for the decomposing of gaseous benzene compared to other controls (Fig. 2b and Table 1). This may provide a new strategy for high-efficiency application of TiO_2_ photocatalyst in building materials field.

In conclusions, based on hierarchical porous magnesian substrate coupling with its chemical *in situ* Ag/AgCl modification method to TiO_2_ particles, the high-activity Ag/AgCl/TiO_2_/PM photocatalytic composite material was explored. The sample revealed excellent photocatalytic removal performance on gaseous benzene. More specifically, Ag/AgCl/TiO_2_/PM with 3.28 × 10^−4 ^wt.% Ag content processed the highest photocatalytic activity for the decomposing of gaseous benzene, its reaction rate (*k *= 2.36 × 10^−2 ^min^−1^) was 5.21 and 30.57 times higher than that of TiO_2_/PM and TiO_2_/imporous magnesian substrate, respectively. The integration synthesis and photocatalysis mechanism of Ag/AgCl/TiO_2_/PM composite material were investigated. The PM substrate which is composed with phase 5 crystals (Mg_3_Cl(OH)_5_·4H_2_O) offered well gas diffusion and light transmittance channels for photocatalysis. Moreover, with the Cl^-^ resource of phase 5 crystals, the Ag/AgCl/TiO_2_/PM composite structure was simultaneously synthesized by *in situ* method, which enhanced the light absorption response and charge transfer properties of TiO_2_ particles. Therefore, the synthesis method and prepared material in this paper provide a novel strategy for high-efficiency application of TiO_2_ photocatalyst in engineering filed.

## Methods

### Chemicals and reagents

Magnesium oxide (MgO, analytically pure, Liaoning Ying kou Co., Ltd), magnesium chloride (MgCl_2_·6H_2_O, analytically pure, Liaoning Ying kou Co., Ltd), bone glue protein foaming agent with solid content of 36%, specific gravity of 1.16g/cm^3^, PH of 9~10, was purchased from Henan Province (Yongtai co. ltd, technical grade), China. Degussa P25 (20% rutile and 80% anatase) with 99.5% purity and 30 nm particle size was used as TiO_2_ photocatalyst. Silver nitrate (AgNO_3_) and absolute alcohol (C_2_H_5_OH) were analytical reagents, which were provided by Shenshi Chem.

### Photoctalytic materials synthesis

**Figure 4:**
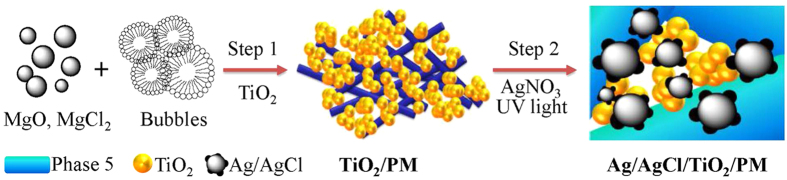
Schematic diagram illustrating the preparation of sample. **Step 1**: preparation of PM substrate; **Step 2**: synthesis of Ag/AgCl/TiO_2_/PM sample.

Porous magnesian (PM) substrate was prepared through a sample method. As shown in Fig. 4 (Step 1), in a typical preparation, 43 g magnesium oxide powder and 57 g MgCl_2_ water solution (w% = 27.4%) were added into 500 mL polytetrafluoroethylene beaker stirring 3 min. Then 200 mL bubbles with (0.04 ± 0.005) g/cm^3^ volume density was added, and stirring 2 min to form PM fresh slurry. The bubbles was prepared with 3 mL foaming agent and 50 mL deionized water by mechanical stirring method (2000 r/min). After that, casted PM fresh slurry into silicone moulds, and aging 28 d in the temperature at (20 ±1) °C and relative humidity at (60 ± 5)%, then the PM sample was demoulded and cut into slices of Φ160*3 mm. Subsequently, the slice was cleaned by compressed air and placed in the oven at 65 °C. The synthesis of Ag/AgCl/TiO_2_/PM sample was achieved by self-assembly and photo-reduction method (Fig. 4 (Step 2)). Firstly, PM substrate was put into vacuum saturation apparatus and loading 0.1 MPa vacuum 30 min at room temperature, 500 mL 1 g/L TiO_2_ absolute alcohol dispersion solution was inhaled into the above container with a stirring to keep the homogeneous suspension solution, after 1 h, TiO_2_/PM precursor was taken out, and washed by distilled water, and then dried in oven at 85 °C. Secondly, a certain amount of AgNO_3_ ethanol-water solution (ethanol/water volume ratio = 3/1, C_AgNO3_ = 5.0 × 10^−3 ^mol/L) was sprayed on TiO_2_/PM surface for 30 min, and then the prepared sample was irradiated under 300 W high pressure Hg lamp (China, Shanghai Yaming, GYZ, λ_nm_ > 340 nm, the light spectrum with peaks around 365, 400, 440, 550 and 580 nm) for 10 min. After that, the resulting sample was washed with 65% ethanol-water solution (volume ratio), and finally dried at 85 °C for 4 h. Ag/AgCl/TiO_2_/PM samples were denoted by mass fraction of Ag to TiO_2_/PM (Ag wt.%), the weight of Ag was calculated by AgNO_3_ spraying amount.

### Characterization and Photocatalytic performance

The crystalline phases of samples were determined by X-ray diffractometer (XRD, PHILIPS P W 3O4O/60X′PertPRO) with a Cu Kα ray source. Environmental Scanning electron microscopy (ESEM, Quanta FEG 450, FEI) together with a Backscattered electron (BSE) detector were used to observe the morphology of samples. X-ray Photoelectron Spectrometer (V. G.Scientific.Ltd; Al K Alpha) test was carried out to obtain the chemical state of element and bonding energy. UV-vis diffuse reflectance spectra were recorded in diffuse reflectance mode (R) and transformed to absorption spectra through the Kubelka-Munk function^29^,^30^. A Lambda 35 (PerkinElmer) spectrometer equipped with a Labsphere RSA-PE-20 integration sphere and MgO as a standard were used. N_2_ adsorption-desorption isotherms were measured on an ASAP 2020 instrument (Micromeritics, USA). The photocatalytic performance was evaluated by removal of gaseous benzene in a closed cylindrical stainless steel gas-phase reactor (2.35 L) with a quartz window. The light source is a 300 W high pressure Hg lamp (China, Shanghai Yaming, GYZ, λ_max_ ≈ 365 nm), the distance between the lamp and the sample surface was 35 cm. 2 μl of benzene liquid was injected in the reactor, and the mixture was irradiated by the light as the liquid benzene completely evaporated into gaseous. The samples were measured every 5 min by gas chromatograph (GC2020, Wuhan, Hengxin, which equipped with a flame ionization detector, a methane converter). For repeated photocatalytic performance test, after each photocatalysis, the photocatalyst was irradiated under UV-C lamp (Philips, 36W, λ_max_ = 254 nm) for 24 h to completely decompose the absorbed gaseous benzene molecules. Then, covered the reactor again, 2 μl of benzene was injected in the reactor, and the Hg lamp was turned on to start the next test. Each procedure was repeated three times and the data were given as the arithmetic mean standard deviation. The degradation rate and reaction rate were calculated by as following formulas:


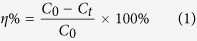



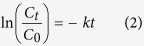


where *C*_*t*_ and *C*_*0*_ are the concentrations of the primal and remaining benzene, *k* is the reaction rate; *t* is the light illumination time.

## Additional Information

**How to cite this article**: Yang, L. *et al.* An *in-situ* synthesis of Ag/AgCl/TiO_2_/hierarchical porous magnesian material and its photocatalytic performance. *Sci. Rep.* **6**, 21617; doi: 10.1038/srep21617 (2016).

## Figures and Tables

**Table 1:** The reaction rate of gaseous benzene removal by various samples.

Sample	Reaction rate (*k*/min^−1^, 90 min)	Half-time (t_1/2_/min)	Relativity (R^2^)
(i)	2.55 × 10^−4^	–	0.9987
(ii)	7.72 × 10^−4^	897.7	0.9893
(iii)	4.53 × 10^−3^	153.0	0.9994
(iv)	7.63 × 10^−3^	90.8	0.9809
(v)	9.78 × 10^−3^	70.9	0.9886
(vi)	1.81 × 10^−2^	38.3	0.9835
(vii)	2.36 × 10^−2^	29.4	0.9727
